# COVID-19 and diabetes—Two giants colliding: From pathophysiology to management

**DOI:** 10.3389/fendo.2022.974540

**Published:** 2022-08-19

**Authors:** Maria Chiara Pelle, Isabella Zaffina, Michele Provenzano, Giovenale Moirano, Franco Arturi

**Affiliations:** ^1^ Department of Medical and Surgical Sciences, University ‘Magna Graecia’ of Catanzaro, Catanzaro, Italy; ^2^ Nephrology, Dialysis and Transplantation Unit, Department of Experimental, Diagnostic and Specialty Medicine (DIMES) Alma Mater Studiorum, University of Bologna, Bologna, Italy; ^3^ Department of Medical Sciences, University of Turin, CPO-Piemonte, Turin, Italy; ^4^ Research Center for the Prevention and Treatment of Metabolic Diseases (CR METDIS), University ‘Magna Graecia’ of Catanzaro, Catanzaro, Italy

**Keywords:** blood glucose, SARS, lifestyle, diabetes mellitus, COVID-19

## Abstract

Since December 2019, a new coronavirus, called severe acute respiratory syndrome coronavirus 2 (SARS-CoV-2), has spread around the world, causing the coronavirus 2019 (COVID-19) pandemic. From the beginning, SARS-CoV-2 has put a strain on the health system. In fact, many patients have had severe forms of the disease with the need for hospitalization due to respiratory failure. To contain the pandemic, the most widely used approach has been lockdowns. Social restrictions have been reduced thanks to the development of vaccines and targeted therapies. However, fatal events still occur among people at high risk of serious infection, such as patients with concomitant diabetes. Different mechanisms have been proposed to explain the poor prognosis of patients with diabetes and COVID-19, but the specific cause is unclear. It is now known that insulin resistance, inflammation, and cytokine storm are involved. Moreover, SARS-CoV-2 uses the angiotensin-converting enzyme 2 receptors to enter cells. This receptor is expressed on pancreatic beta cells and, during infection, it appears that receptor involvement may induce hyperglycemia in patients with or without diabetes. In this study, we discuss the mechanisms underlying the poor prognosis in people with COVID-19 and diabetes and what may improve the outcome in these patients.

## Introduction

On 31 December 2019, an outbreak of pulmonary infection due to an unknown coronavirus (later called severe acute respiratory syndrome coronavirus 2 (SARS-CoV-2)) was first reported in Wuhan, China (1). After a few weeks, the new virus had already spread around the world, quickly reaching pandemic status. In more than 2 years since the onset, there have been about 340 million confirmed cases of coronavirus disease 2019 (COVID-19), and more than five million deaths worldwide. To date, several weapons to use against COVID-19, including vaccines, have been developed. However, fatal events still occur in a nonnegligible proportion of patients, especially among people at high risk of serious infection, such as the elderly or patients with concomitant diabetes mellitus (DM), hypertension, chronic obstructive pulmonary disease (COPD), obesity, and/or cardiovascular disease (2). Indeed, the clinical manifestations of COVID-19 span from completely asymptomatic cases up to severe cases of multiorgan dysfunction. Among the various risk factors that predict adverse outcomes after SARS-CoV-2 infection, DM plays an important role. According to the World Health Organization, about 422 million people worldwide have DM, and there are about 1.5 million DM-related deaths each year, especially in low-middle-income countries. These numbers are expected to increase over time (3). Thus, given the global dimension and burden of DM worldwide, it is important to understand and evaluate the impact of COVID-19 on this population.

DM and COVID-19 co-occurrence embody the perfect example of a syndemic status (4), namely a state in which two or more co-occurring diseases amplify each other in a synergistic manner (5). Generally, DM is a known risk factor for the onset of infections, and published data have shown a greater susceptibility of people with DM to other coronaviruses such as the Middle East respiratory syndrome coronavirus (MERS-CoV) and SARS-CoV-1. In addition, people with DM are often older; have other comorbidities; and have metabolic, pathophysiologic, and immunologic alterations that can create the perfect storm for the onset of severe COVID-19 (6). Considering the global prevalence of DM and the negative impact of COVID-19, in this review, we discuss the mechanisms underlying the poor prognosis in patients with COVID-19 and DM and what may improve the outcome in these patients.

## COVID-19 and DM

DM is a chronic disease associated with microvascular and macrovascular complications. It is one of the main causes of morbidity and mortality in the world (7). Moreover, people with DM have an increased susceptibility to respiratory infections, particularly influenza and pneumonia (8). Consistently, several authors have highlighted that DM is a risk factor for the poor prognosis of COVID-19 (9–11).

Since the beginning of the COVID-19 pandemic, it has been reported that patients with DM have a poor prognosis. The first Chinese report from Wuhan, China, showed that compared with patients without DM, patients with DM had a higher risk of developing complicated infections with pneumonia and acute respiratory distress syndrome (ARDS) and a higher rate of in-hospital mortality (odds ratio (OR) 2.85, 95% confidence interval (CI) 1.35–6.05). As the COVID-19 pandemic continued, other evidence of the poor prognosis of diabetic subjects infected by the SARS-CoV-2 accumulated. For instance, the multicentric French Coronavirus SARS-CoV-2 and Diabetes Outcomes (CORONADO) study involved 1,317 participants with DM, most (88.5%) with type 2 diabetes mellitus (T2DM). The authors reported that 31.1% of participants were admitted to the ICU, including 20.3% who required mechanical ventilation, and 10.6% met the primary outcome, namely death within 7 days of admission. There were no differences between T2DM and type 1 diabetes mellitus (T1DM), likely due to low statistical power (there were only 39 patients with T1DM) (12). In a general population cohort from Sweden, subjects with T2DM were more likely to be hospitalized for COVID-19, as well as admitted to the ICU and die, compared with matched controls from the general population. The excess risk was reduced but persisted after adjustment for comorbidities and other factors only for T2DM (hazard ratio (HR) for COVID-19–related mortality was 1.53, 95% CI 1.39–1.63) (13). Guan et al. (14) reported that the prevalence of DM among Chinese patients with severe diseases was threefold higher than those with nonsevere diseases. In a meta-analysis by Li et al. (15), the prevalence of DM in patients admitted to the ICU was twofold higher compared with those not admitted to the ICU. In a large general population cohort of Scotland (*n* = 5,463,300), McGurnaghan et al. (16) showed that people with T2DM or T1DM had an elevated risk of fatal or critical disease and admission to the ICU. This risk, adjusted for age, sex, and DM duration and type, was higher in those who were men, living in a residential care home, with microvascular complications such as retinopathy and nephropathy, or with poor glycemic control. Moreover, diabetic ketoacidosis (DK) or hypoglycemia that had required hospitalization in the past 5 years increased the risk in these subjects. The use of insulin or sulphonylureas was associated with the highest risks, likely due to the increased risk of hypoglycemia (16) induced by these treatments. A population-based cohort study conducted in the UK showed that mortality in people with T1DM or T2DM had increased during the COVID-19 pandemic (10,989 of 16,743 additional deaths (65.6%)) compared with mortality over the same period in the previous 3 years. Moreover, this study confirmed that beyond classic risk factors (i.e., cardiovascular disease, non-white ethnicity, impaired renal function, age, sex, socioeconomic deprivation, and smoking habit), hyperglycemia and body mass index (BMI) were associated with poor prognosis. In fact, mortality was associated with glycated hemoglobin (HbA1c) >86 mmol/mol (HR 2.23, 95% CI 1.50–3.30, *p* < 0.0001); socioeconomic deprivation (HR 1.93, 95% CI = 1.36–2.72, *p* = 0.0002); and a BMI of <20.0 kg/m^2^ (HR 2.45, 95% CI 1.60–3.75, *p* < 0.0001), 35.0–39.9 kg/m^2^ (HR 1.72, 95% CI 1.21–2.46, *p* = 0.0028), or >40.0 kg/m^2^ (HR 2.33, 95% CI 1.53–3.56, *p* < 0.0001) (6). None of these studies have reported differences in risk by type of diabetes. Barron et al. investigated the relative and absolute risks of in-hospital deaths with COVID-19 by type of diabetes in a population-based study conducted in the UK (17). This study showed an increased risk of death in subjects with diabetes, with a third of all in-hospital deaths with COVID-19 occurring in patients with diabetes. Unadjusted mortality rates were significantly higher for patients with type 2 than for people with type 1 diabetes, with both being significantly higher than for people without diabetes. However, very interestingly, for the first time, adjusted for sex, age, region, and index of multiple deprivation ethnicity, the odds ratio for in-hospital deaths in patients with type 1 diabetes was 3.51 and for patients with type 2 diabetes was 2.03 compared with the population without known diabetes (17). This issue is very important in view of the need for specific advice for people with different types of diabetes and their families.

Several mechanisms have been highlighted to explain why patients with DM have higher mortality and risk of complications than the general population.

**Figure 1:**
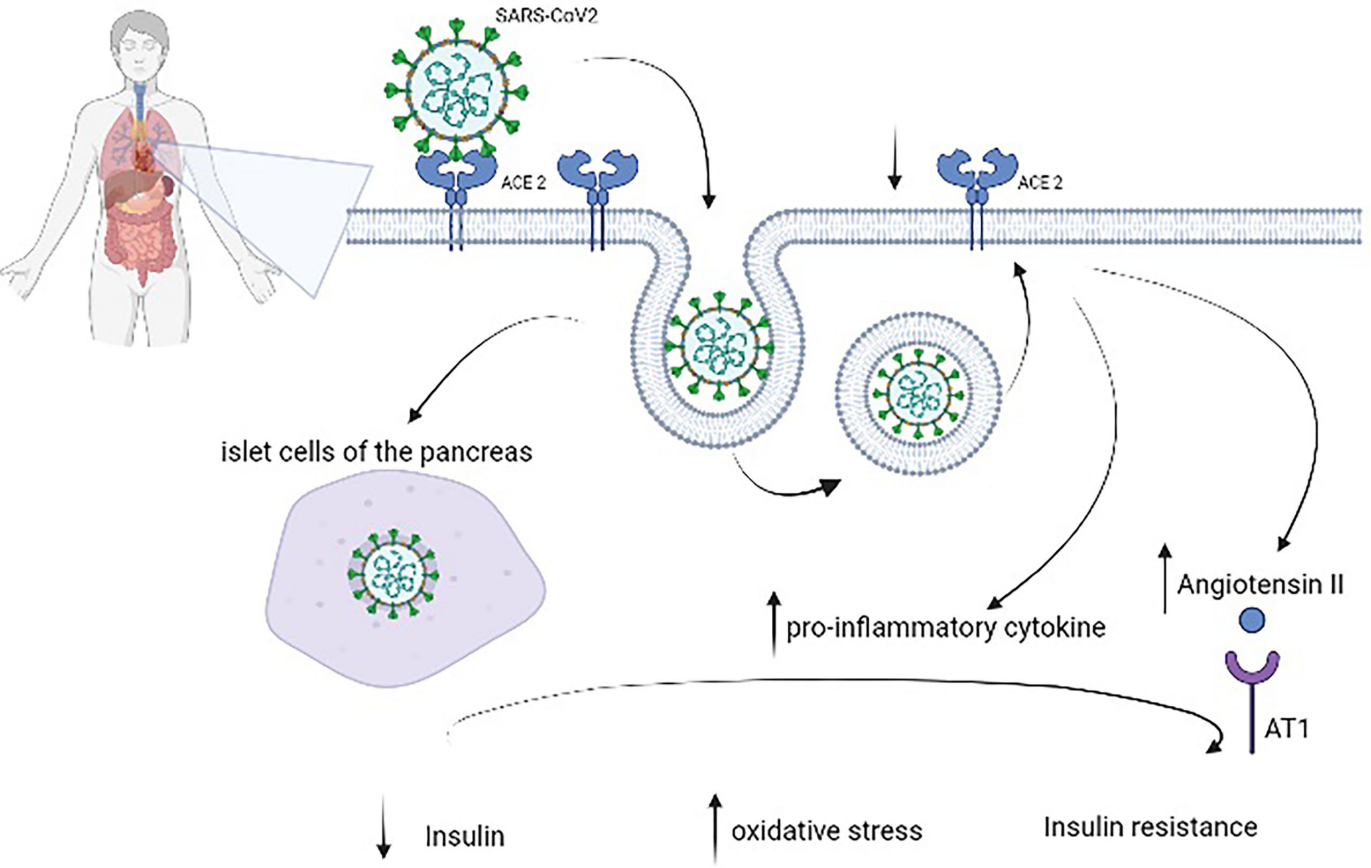
Pathophysiology of diabetes mellitus in patients with coronavirus disease 2019 (COVID-19). Severe acute respiratory syndrome coronavirus 2 (SARS-CoV-2) binds to angiotensin-converting enzyme 2 (ACE2), which is expressed in several organs, including the lungs, heart, kidneys, liver, and stomach, to enter cells. After endocytosis, SARS-CoV-2 could directly damage pancreatic beta cells as well as induce ACE2 downregulation, which leads to overexpression of angiotensin II with its harmful AT1-mediated effects and increases the levels of proinflammatory cytokines. These virus-induced alterations reduce insulin and augment oxidative stress and insulin resistance.

SARS-CoV-2 binds to the angiotensin-converting enzyme 2 (ACE2) receptor, which is involved in several molecular processes and glucose control (18). High plasma glucose levels, defined by two blood glucose measures of >180 mg/dl within a 24-h period, have been related to the risk of mortality in patients with COVID-19 (19). Bode et al. (20) reported a 41.7% mortality rate in their cohort. Zhang et al. (21) confirmed a higher composite outcome risk (mechanical ventilation (MV), admission to the ICU, and death; OR 5.47, 95% CI 1.51–19.82, *p* = 0.010) in patients with hyperglycemia and COVID-19 (defined as fasting plasma glucose (FPG) ≥7.0 mmol/L (≥126 mg/dl) but HbA1c <6.5%) compared to patients with normoglycemia. In addition, ACE2 is expressed in several organs, including the lungs, heart, kidneys, liver, and stomach (22); this could explain the severe disease that evolves into multiorgan failure. Normally, ACE2 is important in the control of inflammation—in fact, it degrades angiotensin II and angiotensin I into smaller peptides, angiotensin-(1–7) and angiotensin-(1–9), respectively. The first has an antioxidant and anti-inflammatory role, through the Mas receptor pathway; this process is altered in patients with DM (23). It seems that in nonsurvivors, there is an imbalance in this pathway with a decrease in angiotensin-(1-7) (24, 25). Moreover, in people with DM, ACE2 expression is increased (26, 27), and it could explain the propensity of these patients to develop severe illness, with ARDS, cardiac involvement, and acute kidney injury. Hyperglycemia is associated with glycation of proteins, microangiopathy of alveolar capillaries, and proteolysis of connective tissue, leading to the collapse of small airways during expiration (28, 29). DM is characterized by low-grade chronic inflammation; in particular, hyperglycemia activates inflammatory pathways and increases oxidative damage, leading to failure of the immune system (**Figure 1**).

In DM, there is an increase in some cytokines, such as tumor necrosis factor-alpha (TNF-α), interleukin (IL)-1β, and IL-8, that facilitate infections (30). Moreover, there is an imbalance between anti-inflammatory and proinflammatory subsets of T cells, with overactivation of the Th_1_ and Th_17_ subsets (31, 32). Moreover, compared with patients with COVID-19 but not DM admitted to the ICU, patients with COVID-19 and DM had significantly higher levels of C-reactive protein (CRP), procalcitonin, ferritin, and IL-6 (33), all of which are involved in a hyperimmune response called a cytokine storm (34, 35). Insulin resistance (IR) and hyperglycemia are involved in endothelial dysfunction and activation of platelet aggregation. Patients with severe COVID-19 and DM also have higher D-dimer blood concentrations (35) and hyperfibrinogenemia (36). This state of hypercoagulability is frequently reported in patients with a poor prognosis due to the formation of microemboli in the lungs and in large arterial vessels, leading to stroke (37).

### Altered FPG as a predictor of poor outcome

When comparing patients with and without DM (38, 39), the FPG level (≥7.0 mmol/l) (40) is an independent predictor of mortality from COVID-19. Moreover, FPG is positively related to the prolonged duration of SARS-CoV-2 clearance (41). Additional studies have demonstrated a relationship between admission FPG level and the degree of severity in patients with COVID‐19; it is also an independent predictor of poor prognosis at 28 days in patients with COVID‐19 (42, 43). Chai et al. (44) reported that FPG ≥7.0 mmol/L at admission in patients with COVID‐19 but not DM was related to chest tightness and slower recovery of lung abnormality 1 year after discharge. Indeed, hyperglycemia promotes the progression of COVID-19 and increases the risk of being admitted to the ICU and the incidence of clinical complications (use of vasoactive drugs or mechanical ventilation) (44). Several hypotheses have been advanced to explain the relationship between FPG and the poor outcome of patients with COVID-19. First, hyperglycemia alters innate cell-mediated immunity; inhibits neutrophil chemotaxis; and reduces phagocytosis by neutrophils, macrophages, and monocytes (41). Indeed, COVID-19 and hyperglycemia are tightly linked. If, as described above, SARS‐CoV‐2 infection impairs glucose metabolism, hyperglycemia could alter the antiviral interferon response, deferring activation of Th1/Th17 cells, inducing oxidative stress, and leading to endothelial dysfunction (44). Data have shown that myeloid cells (monocytes and macrophages) are the most documented in the lungs of patients with COVID-19 and are critical for the pathogenicity of the illness (45). In their *in vitro* study, Codo et al. (46) demonstrated that increased glucose concentrations affect viral replication. They proved that viral load and ACE2 and IL-1β expression increased in SARS-CoV-2–infected monocytes in a glucose-dependent manner. Moreover, they showed that aerobic glycolysis, necessary to produce adenosine triphosphate (ATP), is specifically upregulated in COVID-19-infected monocytes, and it is necessary and sufficient for SARS-CoV-2 replication (46).

## Hyperglycemia and new-onset DM in COVID-19

In patients infected with SARS-CoV-2, it is common to experience “new-onset” hyperglycemia classified as “stress induced,” “new-onset DM” in unknown prediabetes, or “secondary DM” following the use of corticosteroids (47). New-onset DM is defined by the American Diabetes Association (ADA) as two FPG measurements of ≥7.0 mmol/L (≥126 mg/dl) or HbA1c of ≥6.5% or a random glucose level of ≥11.1 mmol/L (≥200 mg/dl) with symptoms of hyperglycemia and without DM in the past. On the other hand, new-onset hyperglycemia without DM is defined as FPG between 5.6 and 6.9 mmol/L (100–125 mg/dl) and/or HbA1c between 5.7% and 6.4% (48). Previous studies have reported that DM is associated with poor prognosis in COVID-19, but COVID-19, in turn, leads to new-onset DM and acute metabolic decompensation of pre-existing DM (15, 40, 49–53).

Muller et al. (54) showed that SARS-CoV-2 infects cells of the human exocrine and endocrine pancreas. Indeed, they demonstrated that beta cells express viral entry proteins (ACE2 and TMPRSS2) and the pancreatic islets have a susceptibility to infection that could be inhibited by the use of remdesivir. Furthermore, they demonstrated that impaired insulin secretion in pancreatic islets, due to infection, is mediated by a glucose-dependent mechanism. Thus, this evidence has supported the concept that the human pancreas is a target of SARS-CoV-2 infection and that this infection induces metabolic dysregulation detected in patients with COVID-19 (54).

Montefusco et al. (55) demonstrated that the COVID-19 inflammatory state, due to a cytokine storm, induced the presence of new-onset hyperglycemia, IR, and beta cell hyperstimulation in patients without a history of DM. They used continuous glucose monitoring to evaluate alterations in glycemic control and showed that normoglycemic patients with COVID-19 had an impaired glycemic profile and greater glycemic variability compared with healthy controls. Moreover, patients treated with tocilizumab showed a significant improvement in glycemic control compared with patients not treated with tocilizumab. Lastly, in the long term, these mechanisms led to beta cell deterioration and worsening of DM due to islet hyperstimulation and glucose toxicity (55).

Several hypotheses have been proposed to understand the pathophysiological mechanism underlying the onset of hyperglycemia or new-onset DM in patients with COVID-19. We will discuss some of these below.

Acute illness is characterized by a relative insulin deficit, increased lipolysis, and free fatty acids (56) that induce stress hyperglycemia due to a cytokine storm. In fact, a cytokine storm is characterized by higher levels of inflammatory markers such as CRP, a higher erythrocyte sedimentation rate, and more white blood cells (15). However, to date, few studies have determined whether stress hyperglycemia is transient or evolves into new-onset DM (57).

SARS-CoV-2 uses ACE2 as its receptor to enter human cells (49, 58, 59). After endocytosis, ACE2 is downregulated, leading to overexpression of angiotensin II. This change, along with direct entry of SARS-CoV-2 into the islet cells of the pancreas, damages beta cells by reducing blood flow. These alterations cause acute beta-cell dysfunction, leading to further impaired glucose homeostasis (60) and delaying insulin secretion (51, 61). SARS-CoV-2 infection promotes oxidative stress in the pancreatic cell (62), resulting in hypoxia and inflammation with impaired glucose metabolism (15). Moreover, SARS-CoV-2 directly damages crucial organs involved in glucose metabolism, such as the kidney and the liver, leading to altered glucose homeostasis (63).

Chronic hyperglycemia downregulates ACE2, which normally has an anti-inflammatory effect, promotes excessive secretion of proinflammatory cytokines such as IL-6 and TNF-α, and stimulates the renin–angiotensin system (RAS), leading to IR (64, 65). IR is distinguished by increased hepatic glucose release, reduced glucose utilization by muscle, and greater lipolysis. In this state, there is a reduced response to insulin (66).

Another hypothesis is that the common administration of dexamethasone in severe COVID-19 worsens the prevalence and severity of hyperglycemia in these patients by steroid-induced abnormalities with the attenuated recovery of beta cell damage (67). The phenomenon favors IR and impaired beta cell function, but the involvement of glucocorticoids to induce hyperglycemia in acute COVID-19 has not been fully elucidated (63).

Lastly, adipose dysfunction is an alternative factor considered to be the cause of hyperglycemia. Indeed, adiponectin and the adiponectin/leptin ratio are noticeably reduced in patients with severe COVID-19 (52).

The tight connection between COVID-19 and DM that has been well described by Mahrooz et al. (68) indicates that the interaction between DM and COVID-19 is a vicious cycle. SARS-CoV-2 infection induces the release of proinflammatory cytokines that, in turn, lead to IR and beta cell dysfunction with a consequent reduction in insulin secretion. Moreover, in patients with DM, SARS-CoV-2 can induce the release of catecholamines and glucocorticoids, which are hyperglycemic hormones that alter glycemic control during metabolic emergencies (DK and hyperosmolar hyperglycemia). All these mechanisms cause hyperglycemia that reduces the immune response and increases the virulence of SARS-CoV-2. Furthermore, the chronic inflammatory nature and the impaired glucose metabolism of DM dysregulate the immune system, including impaired T-cell and macrophage function and neutrophil chemotaxis, thus facilitating SARS-CoV-2 infection (68).

## Lipid homeostasis and effects of SARS-CoV-2 in adipose tissue

Several studies have demonstrated alterations in the serum lipid levels in patients with COVID-19 compared with healthy people. Furthermore, dyslipidemia in these patients can worsen their prognosis. In particular, researchers have reported a significant decrease in the level of high-density lipoprotein cholesterol (HDL-C) only in critical cases and a significant decrease in total cholesterol (TC) and low-density lipoprotein cholesterol (LDL-C) in all patient groups (from mild to severe) (69–71). Li et al. (72) reported that patients with low HDL-C and apolipoprotein A-I (ApoA-I) concentrations at admission had high CRP concentrations, a protracted hospital stay, and augmented disease severity. Moreover, they showed that persistent hypolipidemia, including low TC, HDL-C, LDL-C, and ApoA-I concentrations, was mostly found in patients with COVID-19 who did not survive (72). The exact mechanism to explain these alterations is not well known, but several mechanisms have been proposed. First, in severe COVID-19, arachidonic acid is used to produce cytokines. A cytokine storm can, in turn, induce the release of unsaturated fatty acids as a defense mechanism (73). Additionally, acute inflammation with a high release of proinflammatory cytokines, such as IL-1, IL-6, IL-12, interferon-gamma (IFN-γ), and TNF-α, can reduce the synthesis and/or secretion of apolipoproteins (74–76). Dias et al. (77) demonstrated that SARS-CoV-2 activates reprograming of lipid metabolism in monocytes, increasing the expression of CD36 (involved in lipogenesis), PPARγ, and SREBP-1 24 h after infection. Lastly, SARS-CoV-2 liver dysfunction can impair lipid metabolism, affecting the synthesis of apolipoproteins and lipoprotein in severe COVID-19 (78).

## DM and cardiometabolic multimorbidity

A Finnish population-based study concluded that T2DM is a “coronary heart disease equivalent.” The authors included 1,373 people without DM (638 men and 735 women) and 1,059 people with T2DM (581 men and 478 women). The follow-up period was 18 years, and they investigated the incidence of death due to coronary heart disease (CHD). The authors showed that T2DM without prior myocardial infarction had a similar risk for CHD death when compared with a group with prior myocardial infarction without DM (79). During the COVID-19 pandemic, several studies did not show independent associations between poor outcomes in hospitalized patients with T2DM and with a prior history of cardiovascular events (12, 80, 81). Infection with SARS-CoV-2 increased the risk of hospitalization in patients with cardiovascular disease (82, 83). Moreover, a previous history of CHD increases the risk of in-hospital death for COVID-19 (84). Two Italian studies (CoViDiab I and CoViDiab II) (85, 86) showed that the prevalence of previous cardiovascular events was similar in people with T2DM with and without COVID-19. Moreover, patients with T2DM with other cardiometabolic multimorbidity (defined as two or more DM, hypertension, and dyslipidemia) had a higher risk for ICU admission or death than those without DM. The authors hypothesized that cardiometabolic risk factors increased hypercoagulable and proinflammatory states in patients with DM.

## What can improve the prognosis of patients with DM and COVID-19?

Understanding the distal and proximal causal pathways linking DM to severe COVID-19 is necessary to identify potential interventions and optimal treatments. Considering the close relationship between hyperglycemia and poor outcomes in patients with COVID-19, it is evident that good glycemic control is necessary for a proactive approach in these patients. For example, a recent cohort study underlined that well-controlled blood glucose levels are associated with sensibly lower mortality compared with individuals with poorly controlled blood glucose levels during hospitalization (87). Thus, standard prescriptions, such as a healthy diet, physical activity, and regular glucose and blood pressure monitoring, to prevent or reduce the burden of chronic hyperglycemia should play a primary role in reducing the risk of poor prognosis in patients with COVID-19 and DM. High-intensity exercise programs lower blood glucose, HbA1c, lipid levels (TC, HDL-C, and triglycerides), and blood pressure and improve body composition and endurance in individuals with T2DM (88). Hence, they should represent a key intervention to lower the risk of severe COVID-19 in the diabetic population.

Following a healthy diet is another crucial step to controlling HbA1c, FPG, and the metabolic profile in patients with T2DM. A recent study involving more than 500,000 adults found that following a healthy diet was associated with a 9% lower risk of getting infected by SARS-CoV-2 and a 41% lower risk of developing severe COVID-19 compared with people who reported eating the least fruits and vegetables (89). The authors quantified the diet quality by using the validated healthful plant-based diet index (hPDI score): healthy plant foods (nuts, whole grains, vegetables, fruits, legumes, tea/coffee, and vegetable oils) receive a positive score, while animal foods and fruit juices, sweets/desserts, sweetened beverages, potatoes, and refined grains receive a negative score. Plant-based diets are associated with a substantially lower risk of developing T2DM (90). The low risk of SARS-CoV-2 in people who consume a healthy diet is perhaps explained by the vitamin (A, B_6_, B_12_, C, D, and E), folate, omega 3 fatty acid (docosahexaenoic acid and eicosapentaenoic acid), and polyphenol contents. These components could change the immune response and improve glycemic control.

## Therapeutic approaches in patients with COVID-19 and DM

All patients, particularly those with T1DM, should be instructed to increase the frequency of blood glucose measurements and to recognize the management of DK (91). To reach near-normal blood glucose levels, all available treatments must be put into practice: psychological approaches to a healthy lifestyle, intensive blood glucose monitoring, and reasonable drug treatment (92). Recent recommendations advise that drugs that may induce hypoglycemia should be avoided, and doses of oral medications may need to be reduced (93). Antidiabetic therapy must be started or strengthened for patients who repeatedly have preprandial blood glucose values of >180 mg/dl (>10 mmol/dl). The target preprandial glucose level is usually 140–180 mg/dl. When choosing the drug, it is important to remember that hypoglycemic drugs frequently used to treat DM could affect COVID-19 pathogenesis (94). In patients with T2DM, oral hypoglycemic drugs must be re-evaluated upon admission in relation to disease severity, glucose level, chronic treatment, and other factors that could worsen adverse events such as dehydration and reduced oral intake (93). Below, we briefly explain the effects on inflammation of the various classes of drugs used in DM (**Table 1**).

**Table 1:** Drugs to treat diabetes mellitus in patients with COVID-19: from mechanisms to indications for use.

Antidiabetic medications	Mechanisms	Conclusions from the literature	Indications for use/discontinuation
**Metformin**	1. Activation of AMPK, leading to: - Altered conformation of ACE2, decreasing SARS-CoV-2 binding (95) - Reduction of TNF-α and mTOR inhibition, with consequent anti-inflammatory and immunomodulatory activities (95) 2. Other actions: - Reduced production of ROS, oxidative stress, and DNA injury (96)	Reduced risk of death and hospitalization among patients with COVID-19 and DM (95, 97)	It may be continued in patients with milder forms of COVID-19 (95) Withdraw in patients with severe respiratory distress, renal impairment, or heart failure Risk of lactic acidosis and hypoxemia (95, 98)
**DPP4is**	- Reduction of proinflammatory cytokines (99) - Antifibrotic and immunomodulatory effects (100–105)	Nonhomogeneous data on the reduction of mortality in patients with DM (106)	Useful in patients with mild-to-moderate symptoms (106) Neutral effect on cardiovascular outcomes
**GLP-1Ras**	- Action on ACE2 and Mas receptor pathways (24) - Effect on inflammation and fibrosis (107, 108)	Available results indicate an advantageous effect on hospitalizations and mortality in patients with COVID-19 and DM (109, 110)	Useful in patients with mild-to-moderate symptoms (94)
**SGLT2is**	- Upregulation ACE2 (111) - Increase angiotensin (1-7) (112) - Anti-inflammatory, antioxidative, and antifibrotic effects (112) Reduction of cardiovascular risk factors	Heterogeneous and sparse evidence	In patients with cardiometabolic risk factors, evaluate the possible risk of dehydration, ketoacidosis, and acute kidney injury (98)
**Thiazolidinedione**	Increased ACE2 expression (106) Reduced secretion of proinflammatory cytokines (106) Increased secretion of anti-inflammatory cytokines (106)	Insufficient data	Withdraw in patients with acute diseases with specific contraindications (weight gain, edema, and worsening of heart failure) (113)
**Insulin**	- Downregulation of ACE2 receptors (27) - Positive effect on inflammation and coagulation (114)	- Several studies indicate insulin as the treatment of choice to optimize glycemic control in acutely serious hospitalized patients with COVID-19 (115) - T2DM treated with insulin had a decreased risk of COVID-19 infection requiring hospitalization (85)	Multi-injection insulin therapy or continuous intravenous infusion by a syringe pump in acutely serious hospitalized patients with COVID-19 (116)

ACE2, angiotensin-converting enzyme 2; COVID-19, coronavirus disease 2019; DM, diabetes mellitus; mTOR, mammalian target of rapamycin; ROS, reactive oxygen species; SARS-CoV-2, severe acute respiratory syndrome coronavirus 2; TNF-α, tumor necrosis factor alpha.

### Metformin

In addition to its known antihyperglycemic effect, metformin reduces the risk of death and hospitalization among patients with COVID-19 with DM (117). The mechanism is not clear, but several hypotheses have been proposed. First, it increases the expression and phosphorylation of ACE2, a change that decreases SARS-CoV-2 binding to ACE2 (95). Indeed, AMPK, a target of metformin, acts on the expression and stability of ACE2 (118). Furthermore, it leads to reduced production of reactive oxygen species (ROS), oxidative stress, and DNA damage (96). Finally, metformin diminishes TNF-α (95); reduction in this cytokine is associated with a decrease in mortality, as demonstrated by the use of TNF-α inhibitors (119). In a retrospective study, Li et al. reported that the use of metformin both prior to hospitalization and during treatment in hospital is significantly associated with lower mortality in COVID-19 patients with type 2 diabetes (97). Similarly, treatment with metformin at the time of hospitalization with COVID-19 infection appears to have better outcomes in terms of reduced need for intensive care (95). It is important to note that metformin presents several side effects (120) and potentially life-threatening adverse effects in acute illness, such as lactic acidosis (121) and acute renal disease. Therefore, metformin is not the proper therapy in patients with severe respiratory distress, renal impairment, or heart failure. The evidence supports the use of metformin when COVID-19 infection is not severe (95). In critically ill patients with COVID-19, metformin should be discontinued (95, 98).

### Dipeptidyl peptidase-4 inhibitors and glucagon-like peptide-1 receptor agonists

Because other coronaviruses use DPP4 as a receptor to enter the host cell, researchers have suggested that dipeptidyl peptidase-4 inhibitors (DPP4is) may have a beneficial role against SARS-CoV-2 (122, 123). Based on this hypothesis, Fadini et al. (124) carried out a case-control study comparing DPP4is treatment among patients with COVID-19 and T2DM with the expected rate of DPP4is treatment in similar patients without COVID-19. The authors showed no evidence that DPP4is prevents people with T2DM from becoming infected by SARS-CoV-2. In the CORONADO study, the authors showed no evidence that treatment with DPP4is affects the severity of COVID-19; 21.6% of the population had been treated with DPP4is (12). Furthermore, some evidence suggests that incretin-based therapies may be helpful in people with COVID-19 and DM by improving the course of infection and reducing mortality in patients with DM (125). These hypotheses are based on the fact that DPP4 could affect the regulation of the immune system and increase inflammation (94, 95, 100–105, 117, 118). Moreover, it is unclear whether DPP4 could be a target receptor of SARS-CoV-2, similar to MERS-CoV (126). The use of DPP4is could promote anti-inflammatory activities *via* the reduction of proinflammatory cytokines (99). In a recent study, the administration of DPP4is induced neither harmful nor beneficial effects, so the authors did not advise the discontinuation of this class of drugs (127). In an Italian retrospective case-control study, however, the use of sitagliptin during hospitalization was associated with reduced mortality and better clinical outcomes (125). A retrospective study including patients with a moderate–severe SARS-COV-2 infection showed that treatment with DPP4is had no significant influence on clinical outcomes or mortality (106). To date, the published data do not contraindicate the use of DPP4is in patients with COVID-19 and T2DM (12, 125). However, further research is required to define the exact role of DPP4is in the course of COVID-19 in patients with diabetes.

Glucagon-like peptide-1 receptor agonists (GLP-1Ras) act on ACE2 and Mas receptor pathways, thus potentially preventing SARS-CoV-2 infection (24) and modulating inflammation (107) and fibrosis (108). The role of GLP-1RAs in the treatment of COVID-19 and T2DM is not clear. In a multinational retrospective cohort study, patients in treatment with GLP-1RAs showed a significant reduction in mortality (47%) and hospital admissions (40%), including a reduction in respiratory complications (46%) (109). A recent meta-analysis has also analyzed the impact of preadmission use of GLP-1Ras on the mortality outcomes of COVID-19 among patients with diabetes mellitus. Very interestingly, the data showed that preadmission usage of GLP-1RAs was associated with a reduction in mortality rate in patients with DM and COVID-19, independently of gender, age, gender, cardiovascular disease, hypertension, and the use of metformin and/or insulin (110). However, their initiation in critically ill patients is not fully recommended since they require titration, need time to become effective, and could be associated with several side effects (128). Recently, Lim et al. (94) advised using DPP4is and GLP-1Ras in patients with mild-to-moderate symptoms of COVID-19.

### Sodium-glucose cotransporter 2 inhibitors

Sodium-glucose cotransporter 2 inhibitors (SGLT2is) are antidiabetic drugs that mainly act on the kidneys and inhibit renal glucose reuptake. Several studies have demonstrated that SGLT2is are also able to reduce renal and cardiovascular complications. SGLT2is have been shown to increase the expression of ACE in the kidney, and it is thought that they may increase susceptibility to COVID-19 infection (111). On the other hand, the upregulation of ACE2 leads to an increase in the production of angiotensin-(1-7), a vasodilator with both antioxidative and antifibrotic properties (112). One of the main indications in the therapeutic management in patients with COVID-19 and DM is that hypoglycemic drugs that could lead to volume depletion or hypoglycemia must be discontinued (98). Indeed, dehydration can predispose patients to lactic acidosis and DK during acute illness; thus, SGLT2is must be temporarily withdrawn in hospitalized patients (98, 129). Despite their cardiorenal beneficial effects in cardiovascular outcome trials, the use of SGLT2is might be complicated and even potentially dangerous in patients requiring critical care. However, a recent trial proved the efficacy and safety of dapagliflozin in hospitalized patients with COVID-19 with at least one cardiometabolic risk factor (DARE-19), and SGLT2is were well tolerated and fewer serious adverse events were observed in the dapagliflozin versus placebo group (130). The benefits of SGLT2is treatment during COVID-19 remain unknown, and the possible risk of diabetic ketoacidosis during treatment with gliflozin should not be ignored, and some cautions are necessary (106).

### Sulphonylureas and thiazolidinediones

In current medical practice, hypoglycemic therapy must be at doses sufficient to reach glycemic control while avoiding hypoglycemia (131). In this view, sulphonylureas, which increase the hypoglycemic risk in the presence of reduced oral intake, must be discontinued during acute illness (113). Due to its adverse effects, such as fluid retention, thiazolidinedione must be withdrawn in patients with acute diseases (113). Moreover, this drug class appears to increase the expression of ACE2, thus augmenting the susceptibility of cells to SARS-CoV-2 entry (106). Pioglitazone, an example of thiazolidinediones, has shown anti-inflammatory activity and may reduce the secretion of some proinflammatory cytokines (106).

### Insulin

Several studies indicate insulin as the treatment of choice to optimize glycemic control in acutely serious hospitalized patients with COVID-19 (115). Insulin presents a significant anti-inflammatory effect in critically ill patients (27). Moreover, it seems that insulin could downregulate ACE2 (27). Treatment with insulin leads to optimal glycemia control in patients with T2DM and COVID-19 and seems to have a positive effect on inflammation and coagulation (114).

A case-control study (CoViDiab I) showed that people with T2DM treated with insulin had a decreased risk of COVID-19 infection requiring hospitalization (85). Some evidence has demonstrated that patients with severe COVID-19 and DM have a higher insulin requirement (92, 93), which may be explained by the dysfunction of beta-cells or the high inflammatory process induced by the virus. For these reasons, the best therapeutic choice for these patients is multi-injection insulin therapy or continuous intravenous infusion by syringe pump (116) with adequate enteral nutrition or regular oral food intake. Importantly, the use of insulin has some disadvantages, among which is the need to closely monitor the patient’s glucose levels to avoid hypoglycemia (132, 133). Regarding the resumption of previous therapy, current guidelines suggest that it be resumed at the time of discharge (93).

## Effectiveness and safety of COVID-19 vaccination in people with DM

In the context of the COVID-19 pandemic, vaccines have played a central role in protecting susceptible people (134). DM is known to impair the innate and adaptive immune systems by reducing antibody responses. However, there is conflicting evidence as to whether DM impairs seroconversion following COVID-19 vaccination. Some researchers have tried to compare the antibody response between patients with and without DM. In a retrospective study with a small sample of patients with nonsevere COVID-19 (*n* = 31), Pal et al. (135) showed that patients with DM had not reached seroconversion following COVID-19 vaccination 2 weeks after diagnosis. Data from two Italian studies that involved patients with or without DM revealed that the presence of DM and hyperglycemia did not impair the kinetics and durability of the neutralizing antibody response (136). Conversely, the CAVEAT study demonstrated a lower antibody titer in patients with DM and HbA1c of >7%, indicating that inadequate glycemic control during the postvaccination period could reduce the antibody response (137). On the other hand, in the COVAC-DM study, Sourij et al. (138) reported that in patients with DM, the age and estimated glomerular filtration rate are predictors for an immunological response after COVID-19 vaccination, while HbA1c levels are not. A recent systematic review demonstrated a lower seroconversion rate in patients with DM than in healthy controls after COVID-19 vaccination (134). Nevertheless, the antibody response was robust and persistent. Therefore, all patients with DM should be vaccinated given the high risk and poor prognosis for COVID-19.

## Conclusions

COVID-19 and DM represent a dangerous combination in terms of mortality and hospitalization risk, in both general and high-risk populations. It is clear that these two entities influence each other and act synergistically, through molecular and clinical pathways, to affect the patient’s prognosis. At the same time, and this is an intriguing point, there is ample evidence that some molecular and clinical factors act as prognostic factors (their presence influences a negative prognosis) as well as predictive factors (their control improves the prognosis). This is the case of hyperglycemia: it is a trigger of severe events among people with COVID-19 and DM but, when controlled appropriately, can reduce the risk of poor outcomes (139).

Moreover, patients with COVID-19 and DM provide an excellent opportunity to use a personalized medicine approach. For example, normalizing blood glucose in patients with COVID-19 and DM may require downtitration (or even suspension) of metformin and SGLT2is, which are, according to guidelines, the current first-line treatments aimed at reducing cardiovascular risk in patients with DM alone (140). All these concepts should be kept in mind to ensure the good impact and management of patients with COVID-19 and DM during the current pandemic—until it improves and, hopefully, resolves completely.

## Author contributions

Conceptualization: MCP, IZ, MP, and GM. Methodology: MCP and IZ. Writing—original draft preparation: MCP, IZ, MP, and GM. Writing—review and editing: MP and GM. Supervision: FA. All authors have read and agreed to the published version of the manuscript.

## Conflict of interest

The authors declare that the research was conducted in the absence of any commercial or financial relationships that could be construed as a potential conflict of interest.

## Publisher’s note

All claims expressed in this article are solely those of the authors and do not necessarily represent those of their affiliated organizations, or those of the publisher, the editors and the reviewers. Any product that may be evaluated in this article, or claim that may be made by its manufacturer, is not guaranteed or endorsed by the publisher.

## Abbreviations

ACE2, angiotensin converting enzyme 2; ACEis, angiotensin-converting enzyme inhibitors; ADA, American Diabetes Association; ARDS, acute respiratory distress syndrome; ApoA-I, apolipoprotein A-I; ATP, adenosine triphosphate; BMI, body mass index; CHD, coronary heart disease; CKD, chronic kidney disease; COPD, chronic obstructive pulmonary disease; COVID-19, coronavirus disease-19; CRP, C-reactive protein; DK, diabetic ketoacidosis; DM, diabetes mellitus; DPP4is, dipeptidyl peptidase-4 inhibitors; FPG, fasting plasma glucose; GLP-1Ras, glucagon-like peptide-1 receptor agonists; HbA1c, glycated hemoglobin; HDL-C, high-density lipoprotein cholesterol; HR, hazard ratio; ICU, intensive care unit; IL, interleukin; IR, insulin resistance; LDL-C, low-density lipoprotein cholesterol; MERS-CoV, Middle East respiratory syndrome coronavirus; MV, mechanical ventilation; OR, odds ratio; CI, confidence interval; RAS, renin-angiotensin system; ROS, reactive oxygen species; SARS-CoV-2, severe acute respiratory syndrome coronavirus 2; SGLT2is, sodium-glucose cotransporter 2 inhibitors; T1DM, type 1 diabetes mellitus; T2DM, type 2 diabetes mellitus; TC, total cholesterol; TNF-α, tumor necrosis factor alpha.
